# Born Too Soon: Every Story Counts: Lessons in Ethical, Inclusive Storytelling from Born Too Soon

**DOI:** 10.1186/s12978-025-02068-0

**Published:** 2025-06-23

**Authors:** Mary V. Kinney, Amy Reid, Mercy Juma, Anna Gruending, Olufunke Bolaji, Doris Mollel, Denise Suguitani, Silke Mader, Karen Walker, Marleen Temmerman, Joy Lawn

**Affiliations:** 1 Division of Global Surgery, University of Cape Town Faculty of Health Sciences, Observatory, Western Cape, Cape Town, South Africa; 2 School of Public Health, University of the Western Cape, Bellville, South Africa; 3 Partnership for Maternal, Newborn, and Child Health, Geneva, Switzerland; 4 United Nations World Food Programme, Nairobi, Kenya; 5 Partnership for Maternal, Newborn & Child Health, Paris, France; 6 African Neonatal Association, Kigali, Rwanda; 7 Doris Mollel Foundation, East Africa Preterm Family Network, Dar es Salaam, Tanzania; 8 Brazilian Parents of Preemies’ Association, Porto Alegre, Brazil; 9 Global Foundation for the Care of Newborn Infants, München, Germany; 10 Faculty of Medicine and Health, University of Sydney, Sydney, Australia; 11 Council of International Neonatal Nurses, Sydney, Australia; 12 Centre of Excellence in Women and Child Health, Aga Khan University, Nairobi, Kenya; 13 London School of Hygiene and Tropical Medicine, London, UK

Storytelling is an effective communication strategy for articulating health needs and service demands, enhancing public understanding of complex and stigmatized health issues, and informing policy reform and innovative public health practices [1–4]. Specific to pregnancy and childbirth, storytelling has influenced perceptions, reduced fear, empowered women, complemented educational interventions, contributed to legal and policy change and has enhanced understanding of complex issues and cultural norms through personal experiences [5–7]. The issue of preterm birth remains a global challenge [8], and personal narratives of preterm birth experience are a critical form of advocacy—amplifying families’ literacy and their voices, highlighting systemic gaps, and reinforcing the call to uphold the rights of babies, mothers, families, and health workers [9].

In 2023, the Born Too Soon movement centred people - women, families and communities and healthcare workers – at the heart of the campaign [9]. In the past decade, there has been renewed attention to storytelling in public health, highlighting the need for authentic, inclusive, and ethically grounded narratives, with growing emphasis on representation, ownership, and informed consent [10, 11]. This commentary presents some of the stories shared in the Born Too Soon report and reflects on the people-centred, ethically sound storytelling approach.

## Storytelling process

A dedicated sub-group of the Born Too Soon Advisory Group, established in 2022 and coordinated by the Partnership for Maternal, Newborn, and Child Health (PMNCH), brought together a diverse mix of stakeholders and was co-led by BBC News Africa journalist Mercy Juma, whose expertise informed the ethical and journalistic standards applied throughout the storytelling process.

Collective learning from previous maternal and newborn health initiatives helped shape the Born Too Soon storytelling strategy [12–14], emphasizing: (1) Co-design – a coordinated, inclusive process involving parent groups, professionals, and storytellers from diverse fields; (2) Consent – ethical engagement with transparent use and withdrawal protocols; (3) Concrete messaging – linking stories to advocacy goals; (4) Context – ensuring diversity across regions, incomes, identities, and health outcomes; and (5) Creativity – sharing stories across varied formats and media to extend reach.

Drawing on these lessons, the sub-group established a set of core storytelling principles (Table 1). Given the sensitive nature of preterm birth, ethical storytelling was prioritized through co-designed consent processes that ensured transparency, ownership of stories, and informed future use. Parent support organizations identified families and individuals willing to share their experiences based on our requests.

**Table 1:** Principles for collecting and using stories for the born too soon movement

Agreed principles for overall Born Too Soon storytelling design
• Diversity across the portfolio: Ensure a diversity of stories across regions, resource/wealth, race, health conditions, and health outcomes. Language should not be a barrier and we will work with local partners, as needed, to collect stories. • “New” stories: Identify stories that have not previously been shared with the global maternal and newborn health community (i.e. not featured in major report or campaign previously). The stories do not need to be recent but our preference is to have stories that have not been widely shared previously.
Agreed principles for handling individual stories
• Exploratory process: Before approaching the identified individual, conduct some preliminary research about them (e.g. find others who can vouch for them, check online for any controversial issues, find out if they are able and willing to share their story) • Consent: Ensure the person understands and signs the consent form and has a clear understanding of how stories will be used with the knowledge that they have a right to withdraw at any time. • Conduct a pre-interview: Have a pre-interview with the person to introduce yourself and make them familiar with Born Too Soon and the consent form. Discuss what they are comfortable sharing and if they are willing to be recorded (video, audio) and share photos. • Interview guide: Use and adapt the co-designed interview guide tailored to the specific person being interviewed. • Feedback to person: Enable the person to review and approve any content developed for the Born Too Soon movement before we publish it, e.g. stories in the report, blogs, etc….

## #BornTooSoon stories

Of the 13.4 million preterm babies born yearly, the *Born Too Soon* movement (2023) shared just a few powerful stories to underscore the human experience behind the statistics [9]. The campaign featured ten individual stories that reflected on personal experiences, loss, community, and hope for the future of addressing preterm birth and stillbirth. The ten stories reflect diverse preterm birth experiences—across regions, income levels, outcomes, and roles—highlighting voices of mothers, fathers, health workers, and advocates.

**Fig. 1:**
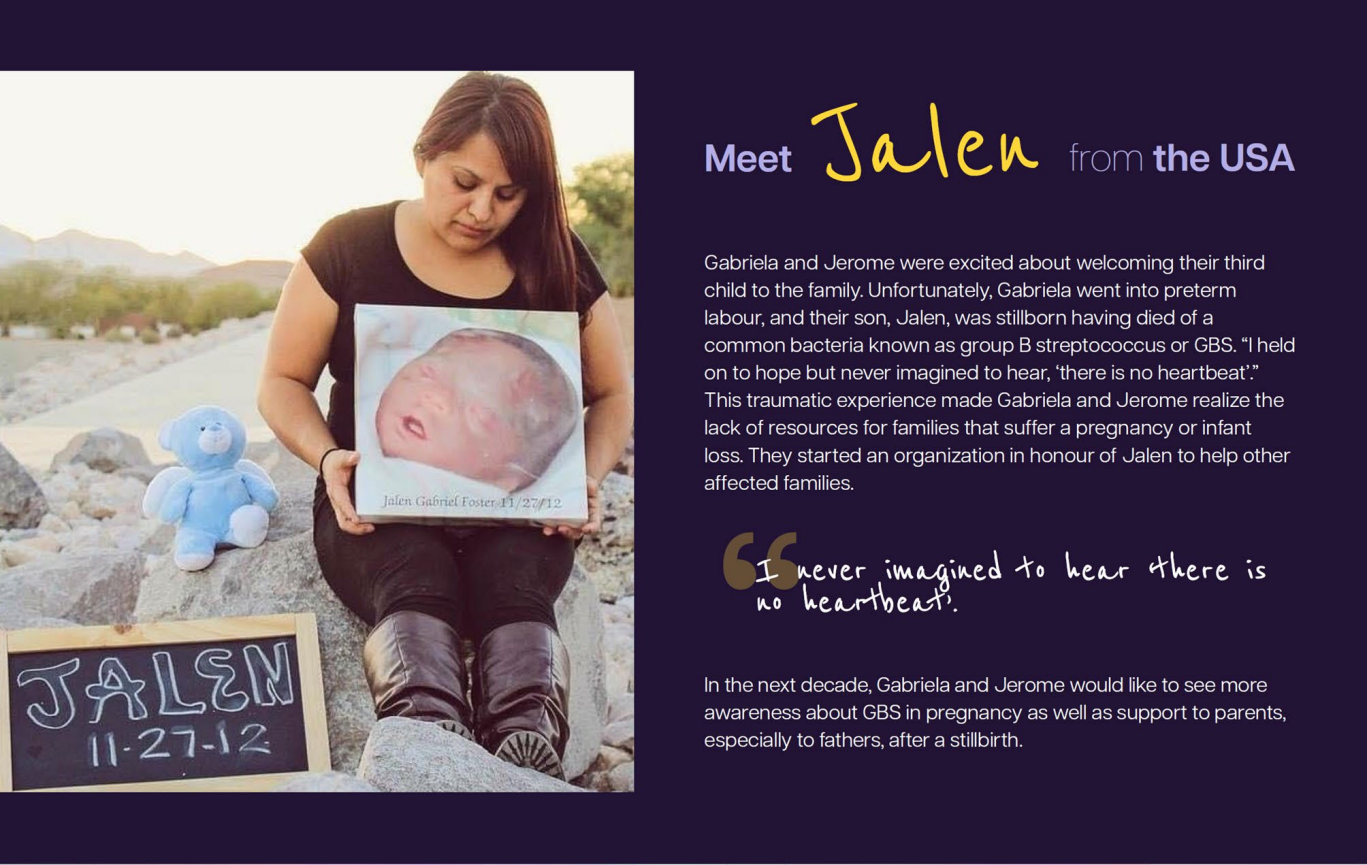
Born Too Soon Stories - Jalen

**Fig. 2:**
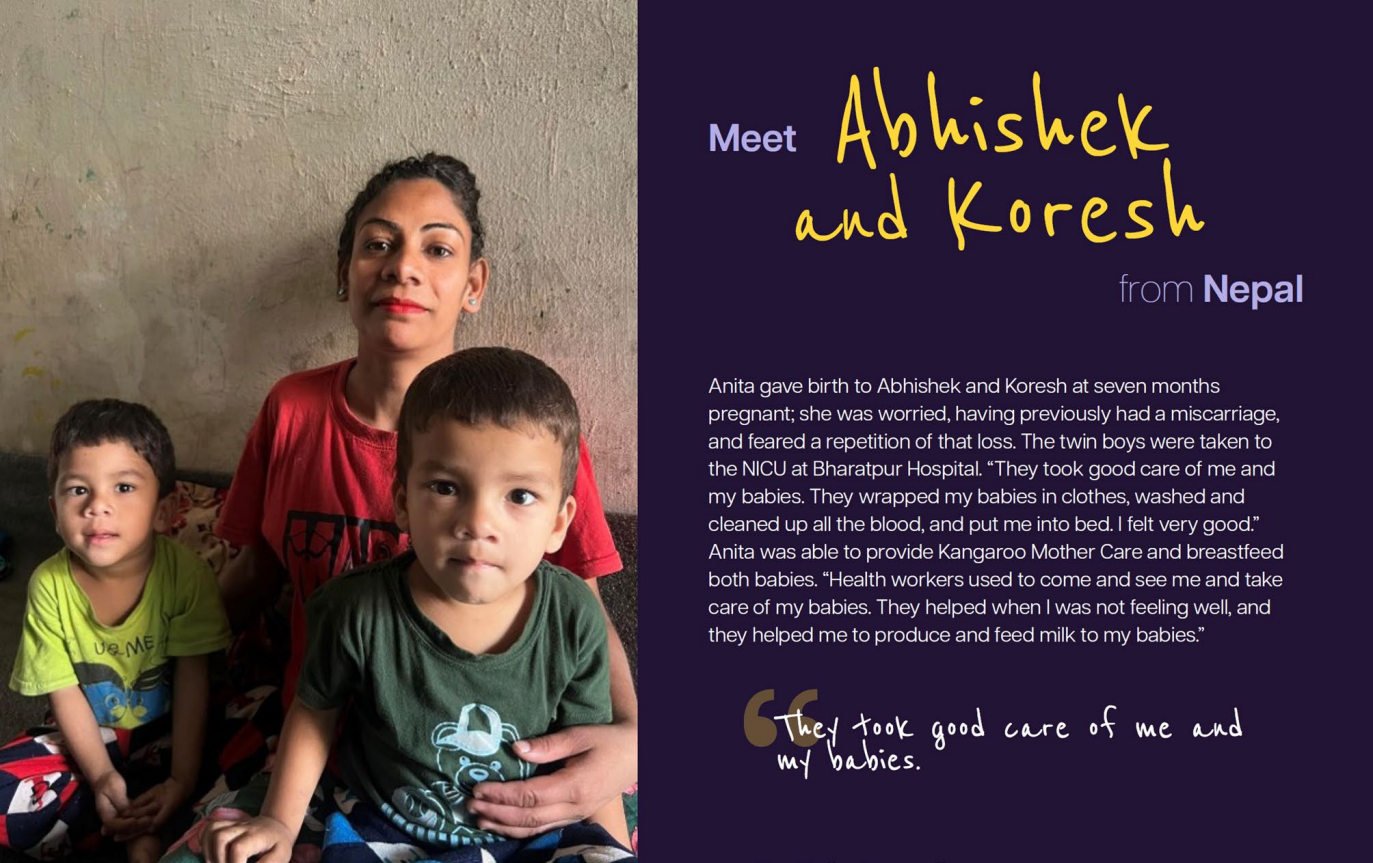
Born Too Soon Stories – Abhishek and Koresh

These stories were disseminated in multiple formats and through multiple channels, including the report, microsite, social media, videos, press outreach, and a photo exhibition called “Behind Every Statistic is a Story - Born Too Soon: An Exhibition” which was unveiled at the launch event in May 2023, and subsequently exhibited at the 76th World Health Assembly [9, 15, 16]. Figures 1 and 2 show two stories featured in the exhibit. Additional stories were gathered as part of the #BornTooSoon Stories and shared through other channels, for example in short videos produced for the report launch.

Some stories highlight the essential role of healthcare workers and the fragile, uncertain early days for families of extremely preterm infants, including stories of affected children Zeek (South Africa), Santiago (Costa Rica), and twins Abhishek and Koresh (Nepal), as well as the story from a health advocate, Chinyere (Nigeria) and Jayme from Japan. Other narratives, including those of Bojidar (Bulgaria), Estelle (Australia), and Ainsley (Kenya), illustrate the emotional toll of inadequate support and emphasize the urgent need for more compassionate, family-centred care [16]. Stories of loss—such as those shared by Gabriela and Jerome Foster (USA) and Staci Shockley (USA)—offered profound insight into the long-lasting grief and trauma experienced by bereaved parents.

Three of these stories are briefly shared below showing the challenges faced and the hopes for the future.

Jayme, born at just 23 weeks and weighing 680 grams, was given little chance of survival. This Born Too Soon story, told by Randal, Jayme’s father tells us how as parents they were devastated by the risks of brain damage and long-term complications facing their newborn son. With intensive medical support, Jayme overcame serious challenges, including underdeveloped lungs and a liver tumor requiring surgery and chemotherapy. Now, a decade later, he is thriving. His parents are inspired by his resilience and hopeful for greater openness and community support around preterm birth, especially in places like Japan where the topic is still rarely discussed.

Ashley Toto felt lost when she became pregnant despite using condoms at age 17. After facing stigma at school and rejection by her family, she left school and moved to Kibera, Nairobi. At 30 weeks, Ashley gave birth to her daughter, Ainsley, who has faced ongoing health challenges. Despite the hardship, Ashley still took her national exams just six weeks after giving birth. Ashley calls for more support for teen mothers and urges society not to dismiss girls who become pregnant early, emphasizing that “it is not the end of life.”

Staci Shockley’s Born Too Soon story tells of her experience with multiple pregnancy losses due to Group B Streptococcus (GBS), losing three pregnancies—including a daughter, a son, and twins—between 2012 and 2019. Each loss followed a routine check-up where no heartbeat was found, and autopsies later confirmed GBS infections in the babies’ hearts, leading to years of grief. By learning more about GBS and working with an infectious disease specialist, she successfully gave birth to healthy twins in 2020. Now a child protection officer, Staci advocates for women to take ownership of their health, urging healthcare providers to listen and respond to women’s concerns.

## Storytelling realities encountered and way forward

The storytelling process in the Born Too Soon movement (2023) effectively merged personal narratives with data, enhancing advocacy efforts for preterm birth. Guided by earlier learnings, the process prioritized inclusivity, ethics, representation, and respect for ownership, the process also introduced new insights.

Here we present five recommendations for future preterm birth storytelling:

1. Plan ahead with sufficient lead time. Although the sub-group was established more than nine months prior to the report launch, this was still a compressed timeline for building trust, ensuring informed consent, and curating stories effectively.
2. Dedicate resources to enable effective coordination. PMNCH played a central role in this by ensuring storytelling was embedded in their workplan for Born Too Soon as an output and the process could receive adequate attention and support.
3. Invest in frontline women’s and parent support groups. Frontline groups played a vital role in identifying stories, building trust with families, and facilitating ethical, respectful engagement. Many of these frontline civil society groups, including women’s health and rights groups, parent support groups, and professional networks, work voluntarily or under major financial constraints. Sustained investment in these groups is essential not only for effective storytelling but also for advancing broader efforts to improve preterm birth outcomes and promote respectful, rights-based care.
4. Establish and nurture equitable and sustained partnerships. Our collaboration brought together diverse stakeholders with different levels of influence—from UN agencies to community-based parent groups – each bringing different regional and institutional perspectives; yet, power imbalances between well-resourced global actors and underfunded local groups may have affected equitable participation. As global partners increasingly rely on civil society for their insights, connections, and storytelling voices, there is a clear need to ensure more equitable participation through flexible tools, transparent processes, and dedicated resources.
5. Embed storytelling as a strategic priority for impact in maternal and newborn health programmes, campaigns, and strategies. Storytelling is not peripheral—it is vital for mobilizing attention and action. Achieving the Sustainable Development Goals related to preterm birth (e.g. ending preventable maternal and newborn mortality and stillbirths, early childhood development, equity) will require stronger focus on voice, dignity, and accountability. Women’s stories related to preterm birth also need to be elevated as they are often intertwined with other health conditions, such as pre-eclampsia or birth trauma. Inclusive, ethical, and effective stories amplify lived experience, shift narratives, and help drive action across diverse audiences, from policymakers to families.

## Conclusion

This commentary highlights the *Born Too Soon* (2023) storytelling approach, showing the power of centering the movement on affected women, families, and health workers. In today’s challenging global health landscape, personal stories bring urgency and clarity, making the case for renewed action on women’s and children’s health and rights. This year, we call for more Born Too Soon stories to be shared as part of the World Health Organization’s 2025 theme of “Healthy Beginnings, Hopeful Futures” [17]. 

Every two seconds, a new story of preterm birth begins. While not all can be told in global advocacy efforts, each story matters and each story has the potential to open minds, move hearts, and shift policy toward a healthier beginning and a hopeful future.

## Data Availability

All data is available in the paper or in supplementary files. Additional information is available at www.borntoosoonaction.org.
